# Sex-dependent association of circulating sex steroids and pituitary hormones with treatment-free survival in chronic lymphocytic leukemia patients

**DOI:** 10.1007/s00277-018-3356-z

**Published:** 2018-05-21

**Authors:** Eric P. Allain, Karin Venzl, Patrick Caron, Véronique Turcotte, David Simonyan, Michaela Gruber, Trang Le, Eric Lévesque, Chantal Guillemette, Katrina Vanura

**Affiliations:** 1 Pharmacogenomics Laboratory, Centre Hospitalier Universitaire de Québec (CHU de Québec) Research Center and Faculty of Pharmacy, Laval University, R4701.5, 2705 Blvd. Laurier, Québec, G1V 4G2 Canada; 2 Division of Hematology and Hemostaseology, Department of Medicine I, Medical University of Vienna, Währinger Gürtel 18-20, 1090 Vienna, Austria; 3 Statistical and Clinical Research Platform, CHU de Québec Research Center, Québec, Canada; 4 CHU de Québec Research Centre, Faculty of Medicine, Laval University, Québec, Canada; 5 Canada Research Chair in Pharmacogenomics, Québec, Canada

**Keywords:** Chronic lymphocytic leukemia, Treatment-free survival, Hormones, Mass spectrometry, Steroids

## Abstract

Chronic lymphocytic leukemia (CLL) is not considered a hormone-regulated cancer although sex is a recognized risk factor with men more frequently diagnosed and developing progressive disease. We hypothesized that variable hormonal exposure may have a sexually dimorphic influence on treatment-free survival (TFS). In 156 CLL cases, we quantitatively profiled 29 circulating steroids (progesterone, adrenal precursors, androgens, estrogens, and catechol estrogens) as well as luteinizing hormone (LH) and follicle-stimulating hormone. Median TFS was shorter for men than that for women (80.7 vs. 135.0 months, *P* = 0.033). Circulating hormone profiles in CLL patients were significantly different from those of healthy donors. In male CLL cases, higher LH levels were associated with shorter TFS (adjusted hazard ratio (HR_adj_) 2.11; *P* = 0.004). In female CLL cases, high levels of the potent androgens testosterone and dihydrotestosterone and the sum of methoxy estrogens were associated with an improved TFS with HR_adj_ values of 0.24 (*P* = 0.007), 0.54 (*P* = 0.023), and 0.31 (*P* = 0.034), respectively. Reduced TFS was observed for women with CLL exhibiting high expression of the steroid-inactivating UGT2B17 enzyme. This study is the first to establish a link between the outcome of CLL patients, sex steroids, and pituitary hormones, revealing a sex-specific hormonal imbalance associated with disease progression.

**Electronic supplementary material:**

The online version of this article (10.1007/s00277-018-3356-z) contains supplementary material, which is available to authorized users.

## Introduction

Chronic lymphocytic leukemia (CLL) is the most common form of adult leukemia characterized by an accumulation and clonal proliferation of mature CD5^+^ B lymphocytes in peripheral blood, bone marrow, and lymphoid organs. A significant clinical and molecular heterogeneity characterizes CLL, leading to vast differences in disease progression, response to treatment, and risk of relapse, with clinical stage, cytogenetic abnormalities, and mutational status of IGHV being the most important prognostic markers [1]. To date, no curative therapy exists aside from allogeneic bone marrow transplantation, however, the development of targeting drugs and their introduction into treatment regimens in recent years portends the prolongation of overall survival (OS) which, depending on clinical stage, lies at a median of 6.5 years [1].

Sex is a risk factor with a significantly different male/female ratio (2:1) in the incidence of CLL that remains unexplained much like for other lymphoid malignancies [2, 3]. Furthermore, men develop progressive disease and resistance to treatment more frequently than women who have better prognosis independently of age and CLL stage, and respond better to therapy [2, 4]. CLL is not considered a hormone-regulated cancer but the sexual dimorphism associated with CLL incidence, prognosis, and response to therapy, in addition to what is known regarding the importance of sex hormones for development and function of the immune system, led to the speculation of a potential protective role for these molecules, most particularly estrogens, in hematologic malignancies [5, 6]. As second explanation, sex-specific somatic alterations in the non-pseudoautosomal and pseudoautosomal regions on chromosomes X and Y have been discussed, [4, 7] however, in-depth data are still lacking.

**Fig. 1:**
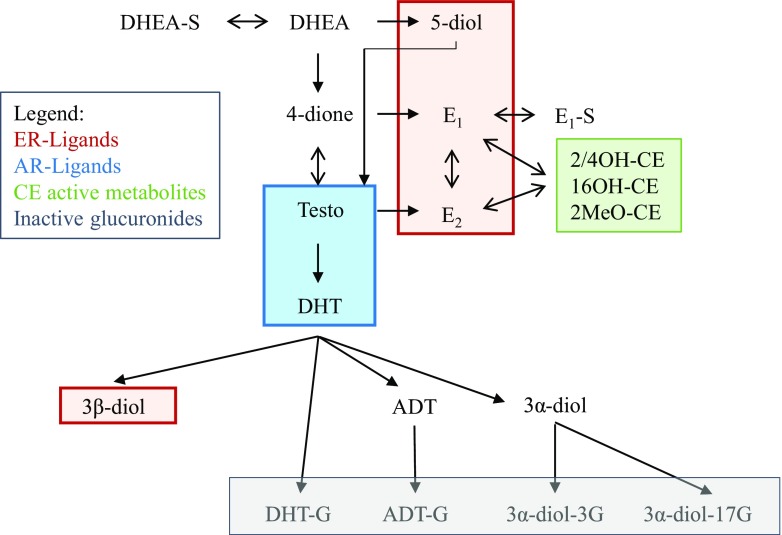
Schematic representation of the steroidogenesis. Major steroid classes are highlighted. DHEA-S, dehydroepiandrosterone sulfate; DHEA, dehydroepiandrosterone; 4-dione, 4-androstenedione; 5-diol, 5-androsten-3β, 17β-diol; Testo, testosterone; DHT, dihydrotestosterone; ADT, androsterone; 3β-diol, androstane-3β-17β-diol; 3α-diol, androstane-3α, 17β-diol; DHT-G, dihydrotestosterone glucuronide; ADT-G, androsterone glucuronide; 3α-diol-17G, androstane-3α, 17β-diol-17-glucuronide; 3α-diol-3G, androstane-3α, 17β-diol-3-glucuronide; E_1_-S, estrone sulfate; E_1_, estrone; E_2_, estradiol; 2/4OH-CE, hydroxy catechol estrogen; 16OH-CE, 16-hydroxy catechol estrogen; MeO-CE, methoxy catechol estrogens; ER, estrogen receptor; AR, androgen receptor

Sex hormones exert their influence via hormone-specific receptors. For estrogens, some studies support the expression of estrogen receptors (ERs) in CLL but with variable results [8–10] with more recent data pointing towards a predominance of ERβ [11]. Hormone receptors like ERβ are ligand-regulated transcription factors activated by naturally produced steroid hormones that regulate transcription of genes controlling a wide variety of biological processes. The endogenous ligands of these hormone receptors comprise numerous steroids produced from cholesterol by the gonads and other organs such as adrenal glands (Fig. 1). Among C19 androgenic steroids, testosterone (Testo) and 5α-dihydrotestosterone (DHT) are the most potent ligands of the androgen receptor (AR), which has been sparingly studied in CLL or other B cell malignancies [12, 13]. For ERs comprised of ERα and ERβ isoforms, many endogenous ligands have been identified including parent C18 estrogens such as estradiol that binds both receptors [14]. Data also indicate that ERα is the major ER isoform expressed in T cells whereas a co-expression of ERβ splice isoforms ERβ1 and ERβ2 was reported in the majority of patients with CLL and normal lymphocytes [10, 11, 15]. Certain C19 steroids including androstenediol (5-diol) and androstanediol (3β-diol), both of which can be synthesized from dehydroepiandrosterone (DHEA), are also highly potent ERβ-ligands [16]. Parent estrogens (E_2_) and estrone (E_1_) may be further converted into diverse catechol estrogen (CE) metabolites that display specific biological activities (mitogenic, antiproliferative, antiangiogenic, pro-apoptotic, and genotoxic properties) [17]. A comprehensive evaluation of this array of hormones in CLL patients is still lacking.

In support of a potential effect of variable hormonal exposure on CLL progression, high expression of the androgen-inactivating UGT2B17 enzyme in peripheral blood mononuclear cells (PBMCs) of untreated CLL patients was associated with reduced treatment-free survival (TFS) and showed promise as a biomarker for IGHV-mutated CLL cases [18, 19]. The *UGT2B17* gene encodes a uridine diphospho-glucuronosyltransferase (UGT) enzyme that conjugates small molecule substrates such as steroids to the polar sugar glucuronic acid, preventing receptor binding, leading to their inactivation and enhanced elimination through bile and urine. UGT2B17 substrates include the potent androgens Testo and DHT, and their metabolites androstane-3α, 17β-diol (3α-diol), and androsterone (ADT) [20]. The *UGT2B17* gene is also highly polymorphic, with a complete gene deletion occurring at varying frequencies (14–92%) depending on ethnicity [21–23]. In healthy donors and cancer patients, altered levels of circulating steroids were reported for individuals with the UGT2B17^null^ genotype (del/del) compared to individuals carrying one or two copies of the gene [24, 25].

To provide a comprehensive evaluation of the potential effect of circulating steroids on CLL prognosis, and most particularly ligands of ERβ, we established their plasma concentrations in a population of female and male CLL patients using mass spectrometry-validated assays, along with androgenic precursors and biologically active estrogen metabolites. We also measured levels of gonadotropins produced by the pituitary gland that act on the gonads including luteinizing hormone (LH) and follicle-stimulating hormone (FSH) by immunoassay. Our primary objective was to examine their relationship to treatment-free survival (TFS) and after adjustment for established prognostic markers. We also studied the influence of *UGT2B17* mRNA expression levels in peripheral mononuclear blood cells (PBMCs) and *UGT2B17* common deletion polymorphism on circulating steroid levels of CLL patients, and their relationship to TFS.

## Methods

### Patients and samples

**Fig. 2:**
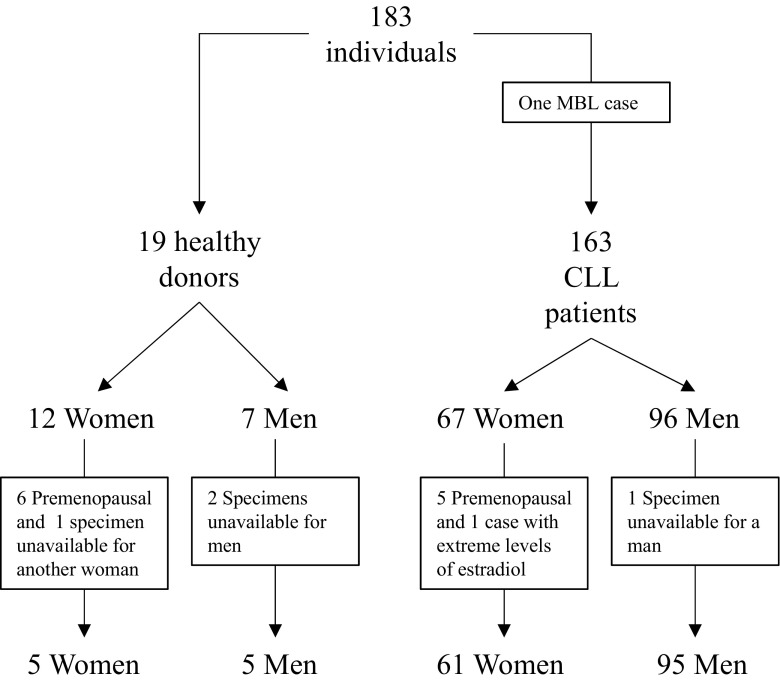
Selection of study population of CLL patients recruited at a single center

In total, 156 CLL patients (61 female and 95 male) diagnosed between 1987 and 2011 at Vienna General Hospital and 10 healthy donors (HD) recruited at the same institution were included (Fig. 2). Patient characteristics were extracted from the clinical records, including sex, age, Binet stage, CD38 expression, Coombs test, cytogenetic aberrations, IGHV gene mutation status and usage, and treatment-free and overall survival. Most patients were early stage and untreated at the time of blood sample collection. CLL diagnosis, staging, and requirement for therapy were based on the NCI-WG2008 guidelines [26]. In addition, 125 HD from a Canadian cohort served as controls [27].

Patients had a median follow-up of 12.75 years and were evaluated for treatment-free survival (TFS), defined as the interval between the date of diagnosis and first therapy. The study was carried out in accordance with the Helsinki Declaration and was evaluated and approved by local Ethical Research Committees of the Medical University of Vienna (ethics vote 1499/2015) and the Centre Hospitalier Universitaire (CHU) de Québec (A14-10-1205).

### Real-time quantitative PCR

Unsorted frozen PBMCs were used for DNA and RNA preparation. Total RNA was analyzed for *UGT2B17* expression using described primers and conditions [18]. Expression values were normalized to the housekeeping control genes Hprt1 and TBP which had been selected according to the qbase procedure [28]. Expression levels were calculated in relation to median healthy donor expression. qPCR data were analyzed using the qbase+ software, version 3.1 (Biogazelle, Zwijnaarde, Belgium - www.qbaseplus.com).

### Mass spectrometry-based quantification of adrenal precursors, androgens, estrogens, and catechol estrogens

Profiling of 29 endogenous steroid molecules was carried out on frozen plasma samples by validated sensitive and specific gas chromatography-mass spectrometry (unconjugated steroids) and liquid chromatography-tandem mass spectrometry (conjugated steroids) by MS multiplex assays to quantify plasma concentrations [25, 27]. Ten unconjugated steroids were measured in a single assay using 250 μl of plasma. Two steroid-sulfates (S) and three steroid-glucuronides (G) were measured in two independent assays using 20 and 100 μl, respectively. The lower limit of quantification (LLOQ; ratio of signal-to-noise ≥ 5:1) was as follows: dehydroepiandrosterone (DHEA; 100 pg/ml); progesterone (50 pg/ml); androstenediol (5-diol) (50 pg/ml); testosterone (30 pg/ml); DHT (10 pg/ml); androsterone (ADT) (50 pg/ml); androstane-3β, 17β-diol (3β-diol) (10 pg/ml); estrone (E_1_) (5 pg/ml); estradiol (E_2_) (1 pg/ml); androstenedione (4-dione) (50 pg/ml); ADT-glucuronide (ADT-G) (1 ng/ml); androstane-3α, 17β-diol 3-G (3α-diol-G) (0.25 ng/ml); 3α-diol-17-G (0.25 ng/ml); DHEA-S (0.075 mg/ml); and E_1_-S (0.075 ng/ml). Only steroid concentrations accurately measured above the lower limits of quantification (LLOQ) were considered as detectable and were reported. All hormones were detected in more than 90% of CLL cases, except 3β-diol (detected in > 65% cases) and progesterone (detected in < 10% cases). All metabolite coefficients of variation (CV) were < 10%.

We also measured 14 catechol estrogens (CE), namely (i) catechol 2OH: 2-hydroxyestrone (2OHE_1_), 2-hydroxyestradiol (2OHE_2_), (ii) catechol 4OH: 4-hydroxyestrone (4OHE_1_), 4-hydroxyestradiol (4OHE_2_), (iii) catechol 16OH: stroll (E_3_), 16α-hydroxyestrone (16αOHE_1_), 16-ketoestradiol (16ketoE_2_), 16-epiestriol (16epiE_3_), and 17-epiestriol (17epiE_3_), and (iv) catechol MeO: 2-methoxyestrone (2MeOE_1_), 2-methoxyestradiol (2MeOE_2_), 2-hydroxyestrone-3-methyl ether (3MeOE_1_), 4-methoxyestrone (4MeOE_1_), and 4-methoxyestradiol (4MeOE_2_). Their quantification was performed by stable isotope dilution LC/MS-MS based on the method published by Xu [29] with some adjustments. Values of catechol estrogens observed below LLOQ were considered as undetected. All CE metabolite coefficients of variation were below 10%.

More details regarding hormone analyses are provided in the Supplementary Material file.

### Statistical analysis

Patient characteristics were portrayed by frequency for categorical variables and median with 95% confidence interval (95% CI) for continuous variables. Clinical and molecular features were compared between men and women, according to *UGT2B17* genotype and expression status, using exact Pearson chi square test. We used a previously published expression threshold for dichotomization of patients into UGT2B17-high and UGT2B17-low expression groups [18]. Hormone levels were presented as means with standard error and were tested between groups with the Wilcoxon-Mann-Whitney test and analyzed as continuous variables. Sex-specific univariate and multivariate analyses of TFS were performed using Cox’s proportional hazard model. The following variables significantly associated with TFS (*P* ≤ 0.05) were included in the adjusted model for both sexes: IGHV, 11q deletion, CD38 expression, and Binet stage. In addition, VH usage and trisomy 12 were associated with TFS for men and women, respectively. Kaplan-Meier survival curves were used to estimate TFS and the log-rank test to compare survival curves. A value of *P* < 0.05 was considered statistically significant. All analyses were performed using SAS version 9.4 and the “survival” package for R version 3.2.2.

## Results

### Characteristics of CLL patients

Cytogenetic markers, IGHV usage, and mutational status as well as clinical parameters are presented for men (*n* = 95) and women (*n* = 61) CLL cases in Table 1. The median age of CLL patients was 59.8 and 62.9 years for men and women (*P* = 0.05), respectively. Most prognostic markers had very similar frequencies between male and female cases with a slightly higher frequency of known prognostic markers in men, although statistical significance was not reached. Statistically significant differences were median treatment-free survival (TFS) time of male patients, which was shorter than that in women (80.7 vs. 135 months, *P* = 0.033) and for the number of patients requiring treatment that was higher in men than in women (64 vs. 27, *P* = 0.008).

**Table 1:** Characteristics of 156 male and female CLL cases

Characteristics	N^a^	Men	Women
Number of patients	156	95	61
Median age (years)	138	**59.8**	**62.9**
Markers			
Binet stage B or C^b^	131	17.5%	7.8%
High CD38 expression^b^	146	31.8%	20.0%
Positive Coombs test	150	2.2%	1.7%
Cytogenetic abnormalities			
17p deletion	97	6.7%	5.4%
11q deletion^b^	149	15.4%	19.0%
Trisomy 12^b^	149	11.0%	8.6%
13q deletion	149	58.2%	51.7%
14q aberrations	149	14.3%	12.1%
Unmutated IGHV^b^	134	50.1%	40.4%
IGHV gene usage^b^			
1–69	114	16.2%	14.6%
3–21	114	4.1%	4.9%
3–23	114	8.1%	4.9%
Treatment-free survival (TFS)			
Median (months)^c^	156	**80.7**	**135.0**
Patients requiring treatment	156	**67.4% (64)**	**44.3% (27)**
Overall survival (OS)			
30th percentile (months)^c^	156	161.2	**191.1**

Significant differences (*P* < 0.05) are highlighted in bold , based on exact Pearson chi square test (men vs. women)

*TFS* treatment-free survival

^a^For each characteristic, the number (*N*) of available individual data is indicated

^b^Variables significantly associated with TFS in both sexes were IGHV, 11q deletion, CD38 expression, and Binet stage. In men and women, respectively, VH usage and trisomy 12 were further associated with TFS

^c^Survival was estimated using the Kaplan-Meier method and comparisons were done using log-rank test. For OS, the 30th percentile is reported in lieu of the median since there were too few events to estimate median survival

### Levels of hormones in CLL patients differ by sex and from those of healthy donors

Hormone levels below the limit of quantification and detected in less than 10% of cases were excluded from the subsequent analysis. Levels of unconjugated steroids and glucuronide or sulfate conjugates differed significantly between men and women, with men presenting significantly higher concentrations of nearly every hormone, except for DHEA, which were comparable between sexes (Supplementary Material: Supplementary Table 1). The most potent androgens and estrogens displayed the greatest disparities, with Testo, DHT, and E_2_ being 15.1-fold, 8-fold, and 4.9-fold more abundant in men compared to women (all *P* values < 0.01). Accordingly, the sums of mean levels of ERβ-ligands (E_1_, E_2_, 3β-diol, and 5-diol) and AR-ligands (Testo and DHT) diverged with 1.6 and 14.3-fold greater levels in men than those in women (all *P* values < 0.01).

**Fig. 3:**
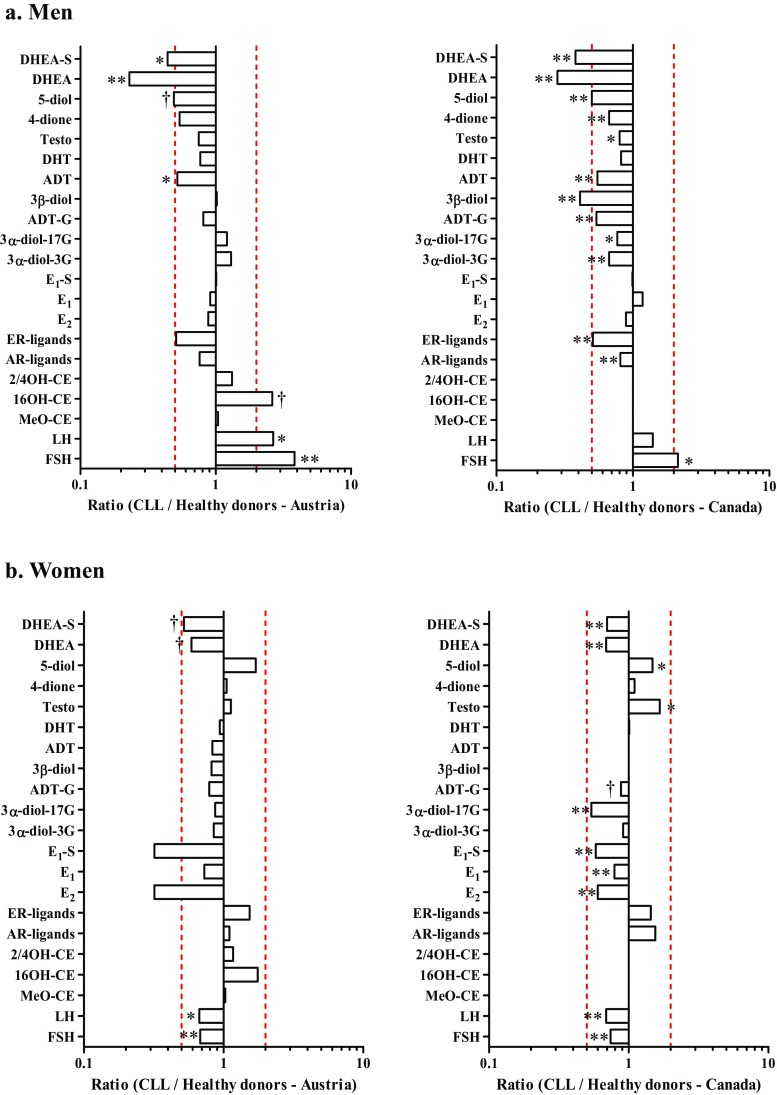
Hormonal imbalances observed in men and women CLL patients compared to healthy donors. Ratios correspond to CLL cases vs. healthy donors for each hormone. Left panels, CLL cases are compared to a limited number of 10 healthy donors (5 men and 5 women), recruited at the same institution (Austria). Right panels, CLL cases are compared to a larger set of (**a**) 15 healthy male and (**b**) 110 healthy female donors (Canada) [27] . Red lines represent a 2-fold change in hormone levels. ^†^*P* < 0.10; *P < 0.05; **P < 0.01, based on Mann-Whitney-Wilcoxon test

We then explored differences in blood levels of steroids between CLL patients and a limited number of healthy donors recruited at the same institution (Fig. 3; Supplementary Material: Supplementary Tables 2, 3). When compared to 10 healthy donors, with an equal number of men and women, male CLL patients had lower steroid levels with significant differences noted for DHEA-S, DHEA, and ADT; female CLL cases showed a trend towards lower DHEA-S and DHEA levels (Fig. 3a, left panel). Men with CLL presented with significantly higher levels of LH and FSH compared to healthy donors, whereas female cases had lower LH and FSH levels compared to healthy women (Fig. 3b, left panel). To further sustain these observations, we also compared CLL patients to a larger number of healthy donors for whom steroid levels were measured by the same MS approaches [27]. For men, most adrenal precursors and androgens were significantly less abundant in CLL cases compared to 15 healthy donors (Fig. 3a, right panel) except for estrogens that were not significantly different and FSH levels that were higher. Compared to healthy postmenopausal women (*n* = 110), significantly lower levels of adrenal precursors (DHEA, DHEA-S), estrogens (E_1_, E_2_, and E_1_-S), and LH and FSH were detected in female CLL cases whereas levels of Testo and 5-diol were significantly higher (*P* < 0.05; Fig. 3b, right panel). Data for catechol estrogens in the larger subset of healthy subjects were not available.

### Treatment-free survival is associated with levels of circulating hormones

**Fig. 4:**
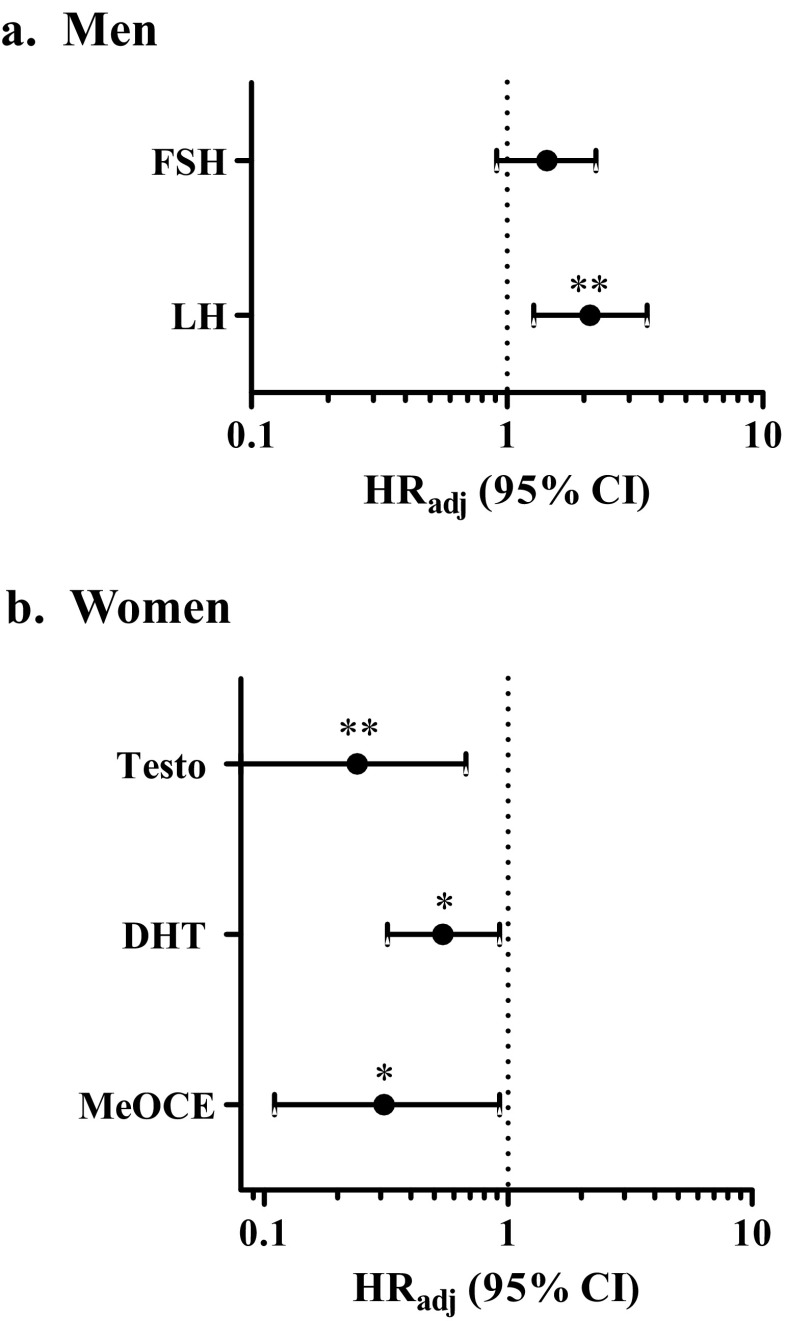
Forest plots for the association of hormones with treatment-free survival (TFS). Adjusted hazard ratio (HR_adj_) with 95% confidence interval for hormones in (**a**) men and (**b**) women CLL patients, calculated with the Cox proportional hazards model. Adjusted models were corrected for IGHV mutation, CD38 expression, Binet stage, 11q deletion in both sexes and further adjusted for VH usage in men, and trisomy 12 in women. LH, luteinizing hormone; FSH, follicle-stimulating hormone; DHEA, dehydroepiandrosterone; 4-dione, 4-androstenedione; Testo, testosterone; DHT, dihydrotestosterone; MeO-CE, methoxy catechol. *P < 0.05; **P < 0.01

In uni- and multivariate analyses, men with CLL displayed no significant differences in TFS based on high or low steroid levels (Table 2). In contrast, higher FSH and LH levels were significantly associated with shorter TFS (univariate: FSH HR 1.42; *P* = 0.028; multivariate: LH-adjusted hazard ratio (HR_adj_) of 2.11; *P* = 0.004) (Table 2; Fig. 4a).

**Table 2:** Treatment-free survival (TFS) based on circulating hormone levels for men with CLL

Plasma steroid levels	HR^c^ (95% CI) (*n* = 95)	HR_adj_^d^ (95% CI) (*n* = 84)	HR_adj_^e^ (95% CI) (*n* = 84)
Adrenal precursors			
DHEA-S (μg/ml)	1.02 (0.80–1.31)	0.87 (0.63–1.19)	0.86 (0.63–1.19)
DHEA (ng/ml)	0.94 (0.67–1.31)	0.86 (0.60–1.12)	0.87 (0.61–1.23)
5-Diol (pg/ml)	0.93 (0.71–1.21)	0.95 (0.72–1.25)	0.96 (0.73–1.26)
Androgens			
4-Dione (ng/ml)	0.89 (0.55–1.44)	1.01 (0.60–1.72)	1.03 (0.61–1.75)
Testo (ng/ml)	1.14 (0.79–1.66)	0.84 (0.53–1.33)	0.85 (0.53–1.34)
DHT (pg/ml)	1.20 (0.88–1.64)	0.96 (0.68–1.37)	0.96 (0.67–1.35)
ADT (pg/ml)	1.09 (0.76–1.58)	1.05 (0.66–1.67)	1.04 (0.66–1.65)
3β-Diol (pg/ml)	0.92 (0.68–1.25)	0.88 (0.60–1.29)	0.88 (0.60–1.29)
ADT-G (ng/ml)	1.22 (0.88–1.70)	*1.01 (0.70–1.47)*	*1.02 (0.70–1.48)*
3α-Diol-17G (ng/ml)	1.14 (0.88–1.46)	0.71 (0.50–1.02)	0.72 (0.50–1.03)
3α-Diol-3G (ng/ml)	1.12 (0.77–1.61)	0.87 (0.57–1.33)	0.87 (0.57–1.32)
Estrogens			
E_1_-S (ng/ml)	1.06 (0.80–1.40)	0.79 (0.58–1.08)	0.78 (0.57–1.07)
E_1_ (pg/ml)	*0.61 (0.35–1.08)*	1.00 (0.51–1.99)	1.01 (0.51–2.00)
E_2_ (pg/ml)	0.86 (0.60–1.22)	0.77 (0.52–1.14)	0.76 (0.51–1.13)
Receptor ligands^a^			
ER-ligands (pg/ml)	0.92 (0.66–1.29)	0.93 (0.67–1.30)	0.94 (0.67–1.31)
AR-ligands (ng/ml)	1.14 (0.78–1.67)	0.84 (0.53–1.33)	0.84 (0.53–1.34)
Catechol estrogens (CE)^b^			
2/4OH-CE (pg/ml)	*0.77 (0.57–1.04)*	0.80 (0.54–1.20)	0.78 (0.52–1.17)
16OH-CE (pg/ml)	*1.33 (0.95–1.85)*	1.32 (0.86–2.02)	1.32 (0.86–2.02)
MeO-CE (pg/ml)	*1.56 (0.92–2.65)*	1.27 (0.70–2.32)	1.28 (0.65–2.49)
Pituitary gonadotropins			
LH (mIU/ml)	*1.35 (0.97–1.89)*	**2.11 (1.27–3.53)**	**2.11 (1.26–3.53)**
FSH (mIU/ml)	**1.42 (1.04–1.94)**	1.43 (0.91–2.23)	1.42 (0.91–2.23)

Significant HR (*P* < 0.05) are highlighted in bold, trends (*P* < 0.10) are in italics. Sex hormone levels were analyzed as continuous variables. Hormone levels in univariate analyses were available for all 95 male patients, except for catechol estrogens and gonadotropins (83/95). Co-variable data for multivariate analyses were available for 84 patients, except for catechol estrogens and gonadotropins (73/84)

*ER* estrogen receptor, *AR* androgen receptor, *CE* catechol estrogens

^a^ER-ligands correspond to the sum of E_1_, E_2_, 5-diol, and 3β-diol; AR-ligands corresponds to the sum of Testo and DHT

^b^2/4OH-CE corresponds to the sum of 2OHE_1_ and 4OHE_1_. 16OH-CE corresponds to the sum of E_3_, 16epiE_3_, 16ketoE_2_, and 16αOHE_1_. MeO-CE corresponds to the sum of 2MeOE_1_ and 4MeOE_1_

^c^Univariate Cox regression model

^d^Multivariate Cox regression models adjusted for IGVH status, CD38 expression, Binet stage, 11q deletion, and VH usage

^e^Multivariate Cox regression models adjusted for IGVH status, CD38 expression, Binet stage, 11q deletion, VH usage, and UGT2B17 mRNA expression

Conversely, in women with CLL, high levels of potent androgens including Testo and DHT were significantly associated with improved TFS by 126.8 and 163.3 months with HR_adj_ values of 0.24 (*P* = 0.007) and 0.54 (*P* = 0.021), respectively (Table 3; Fig. 4b). Accordingly, AR-ligands were significantly associated with an improved TFS (HR_adj_ = 0.27; *P* < 0.01) and ER-ligands showed the same tendency (HR_adj_ = 0.59; *P* < 0.10) in female cases. In addition, we observed that high levels of MeO-CE were significantly associated with an improved TFS with a HR_adj_ value of 0.31 (*P* = 0.034) (Table 3; Fig. 4). These associations remained statistically significant upon correction for expression of the *UGT2B17* gene, encoding an androgen-inactivating enzyme and previously associated with poor prognosis in CLL patients [18, 19]. Results were also comparable in a series of analyses with sex hormone variables dichotomized at their median values into high and low groups (not shown).

**Table 3:** Treatment-free survival (TFS) based on circulating hormone levels for women with CLL

Plasma steroid levels	HR^c^ (95% CI) (*n* = 61)	HR_adj_^d^ (95% CI) (*n* = 44)	HR_adj_^e^ (95% CI) (*n* = 44)
Adrenal precursors			
DHEA-S (μg/ml)	0.81 (0.54–1.20)	0.95 (0.51–1.78)	0.95 (0.49–1.82)
DHEA (ng/ml)	**0.63 (0.41–0.97)**	0.68 (0.33–1.41)	0.62 (0.27–1.42)
5-Diol (pg/ml)	0.95 (0.69–1.30)	*0.64 (0.41–1.01)*	*0.64 (0.40–1.01)*
Androgens			
4-Dione (ng/ml)	**0.31 (0.15–0.65)**	*0.34 (0.10–1.16)*	*0.25 (0.06–1.02)*
Testo (ng/ml)	**0.35 (0.20–0.77)**	**0.24 (0.08–0.67)**	**0.24 (0.08–0.67)**
DHT (pg/ml)	**0.47 (0.31–0.72)**	**0.54 (0.32–0.92)**	**0.53 (0.31–0.91)**
ADT (pg/ml)	*0.70 (0.48–1.01)*	0.79 (0.44–1.43)	0.76 (0.40–1.46)
3β-Diol (pg/ml)	0.70 (0.43–1.14)	0.75 (0.31–1.79)	0.74 (0.31–1.80)
ADT-G (ng/ml)	**0.51 (0.31–0.83)**	0.83 (0.40–1.71)	0.81 (0.37–1.75)
3α-Diol-17G (ng/ml)	0.65 (0.38–1.11)	1.09 (0.56–2.12)	1.09 (0.56–2.13)
3α-Diol-3G (ng/ml)	0.73 (0.46–1.18)	0.90 (0.49–1.67)	0.89 (0.47–1.71)
Estrogens			
E_1_-S (ng/ml)	0.82 (0.51–1.33)	1.11 (0.57–2.16)	1.12 (0.57–2.17)
E_1_ (pg/ml)	0.63 (0.37–1.05)	1.16 (0.53–2.57)	1.17 (0.52–2.62)
E_2_ (pg/ml)	0.84 (0.65–1.01)	0.61 (0.30–1.23)	0.60 (0.30–1.23)
Receptor ligands^a^			
ER-ligands (pg/ml)	0.92 (0.62–1.35)	*0.59 (0.34–1.03)*	*0.59 (0.33–1.04)*
AR-ligands (ng/ml)	**0.37 (0.19–0.73)**	**0.27 (0.10–0.70)**	**0.26 (0.10–0.70)**
Catechol estrogens (CE)^b^			
2/4-OH-CE (pg/ml)	0.80 (0.49–1.28)	0.81 (0.46–1.44)	0.68 (0.33–1.41)
16OH-CE (pg/ml)	0.97 (0.65–1.43)	0.86 (0.38–1.94)	0.87 (0.39–1.95)
MeO-CE (pg/ml)	*0.52 (0.26–1.04)*	**0.31 (0.11–0.92)**	**0.19 (0.05–0.69)**
Pituitary gonadotropins			
LH (mIU/ml)	0.81 (0.46–1.42)	1.34 (0.43–4.18)	1.35 (0.44–4.18)
FSH (mIU/ml)	1.18 (0.45–3.07)	1.10 (0.25–4.89)	1.10 (0.25–4.88)

Significant HR (*P* < 0.05) are highlighted in bold, trends (*P* < 0.10) are in italics. Sex hormone levels were analyzed as continuous variables. Hormone levels in univariate analyses were available for all 61 female patients, except for catechol estrogens and gonadotropins (51/61). Co-variable data for multivariate analyses were available for 44 patients, except catechol estrogens and gonadotropins (35/44)

*ER* estrogen receptor, *AR* androgen receptor, *CE* catechol estrogens

^a^ER-ligands correspond to the sum of E_1_, E_2_, 5-diol, and 3β-diol; AR-ligands corresponds to the sum of Testo and DHT

^b^2/4OH-CE corresponds to the sum of 2OHE_1_ and 4OHE_1_. 16OH-CE corresponds to the sum of E_3_, 16epiE_3_, 16ketoE_2_, and 16αOHE_1_. MeO-CE corresponds to the sum of 2MeOE_1_ and 4MeOE_1_

^c^Univariate Cox regression model

^d^Multivariate Cox regression model adjusted for IGVH status, CD38 expression, Binet stage, 11q deletion, and trisomy 12

^e^Multivariate Cox regression model adjusted for IGVH status, CD38 expression, Binet stage, 11q deletion, trisomy 12, and UGT2B17 mRNA expression

### UGT2B17 mRNA expression in peripheral mononuclear blood cells is associated with TFS

In men and women with CLL, high *UGT2B17* mRNA expression in PBMCs was associated with the adverse prognostic marker unmutated IGHV, and with high CD38 expression in female cases (Table 4). We further observed that high expression of *UGT2B17* was significantly associated with shorter TFS in CLL cases with a median of 75.5 months for cases with high *UGT2B17* expression compared to 126.3 months for cases with low *UGT2B17* expression (*P* < 0.01) (Supplementary Material: Supplementary Table 4). Upon stratification by sex, this association remained significant in female cases with a median TFS of 74.1 months in those with high *UGT2B17* vs. 225.9 months for low *UGT2B17* (*P* < 0.01), which was slightly shorter than median TFS in men (80.7 months). Hence, male CLL cases with high *UGT2B17* expression required treatment more than cases presenting low *UGT2B17* expression. The absence of the *UGT2B17* gene (UGT2B17^null^ genotype) tended to be associated with an improved survival in female patients compared to carriers of at least one copy of the *UGT2B17* gene (median TFS of 254 vs. 126 months; *P* < 0.10), a difference not observed in men with CLL (Supplementary Material: Supplementary Table 5).

**Table 4:** Association of high or low *UGT2B17* mRNA expression in peripheral mononuclear blood cells (PBMCs) with prognostic markers and treatment-free survival (TFS) in CLL patients

		Men (*n* = 81)		Women (*n* = 51)^e^		
Characteristics	N^a^	UGT2B17-high	UGT2B17-low	*N* ^a^	UGT2B17-high	UGT2B17-low
		% (39)	% (42)		% (27)	% (24)
Markers^b^						
Binet stage B or C	66	16.1%	17.1%	42	13.0%	5.3%
High CD38 expression	77	39.5%	25.6%	46	**37.5%**	4.5%
Positive Coombs test	79	2.6%	2.4%	48	0.0%	4.5%
Cytogenetic abnormalities^b^						
17p deletion	49	5.0%	6.9%	30	12.5%	0.0%
11q deletion	77	22.9%	9.5%	49	*29.6%*	4.5%
Trisomy 12	77	11.4%	11.9%	49	11.1%	45.5%
13q deletion	77	48.6%	69.0%	49	51.9%	4.5%
14q aberrations	77	11.4%	19.0%	49	11.1%	18.2%
Unmutated IGHV	74	**69.4%**	28.9%	41	**66.7%**	11.8%
IGHV gene usage						
1–69	61	23.3%	9.7%	36	22.7%	7.1%
3–21	61	0.0%	6.5%	36	9.1%	0.0%
3–23	61	3.3%	16.1%	36	4.5%	7.1%
Survival analysis^b^						
TFS, median (month)^c^		*80.7*	*87.8*		*74.1*	*126.3*
Requiring treatment		76.9%	57.1%		59.3%	36.4%
Survival analysis (all cases)^d^						
TFS, median (month)^c^		80.7	87.2		**74.1**	**225.9**
Requiring treatment		76.9%	60.7%		*59.3%*	*50.0%*

Significant differences (*P* < 0.05) are highlighted in bold, trends (*P* < 0.10) are in italics. Based on exact Pearson chi square test (high vs. low). No significant differences in *UGT2B17* expression were noted for CLL cases carrying one or two gene copies of the *UGT2B17* gene (not shown)

^a^For each characteristic, the number (*N*) of available individual data points is given

^b^Patients with the *UGT2B17*^*del/del*^ null genotype (*n* = 14/95 male and *n* = 9/61 female cases) were excluded from analyses based on *UGT2B17* mRNA expression as they do not carry the *UGT2B17* gene and are negative for *UGT2B17* expression

^c^Calculated using the Kaplan-Meier Method

^d^Patients with the *UGT2B17*^*del/del*^ null genotype were included in the UGT2B17-low group

^e^*UGT2B17* expression was not available for one woman

Despite the limited number of patients, we observed that the absence of the *UGT2B17* gene in female patients (UGT2B17^del/del^ genotype; *n* = 9) was associated with significantly lower levels of a direct product of the UGT2B17 enzyme, 3α-diol-17G (*P* < 0.05; Supplementary Material: Supplementary Table 6). In male cases with the UGT2B17^del/del^ genotype (*n* = 14), a trend towards increased levels of Testo and 4-dione by 26 and 19% respectively was observed (*P* < 0.10), consistent with a reduced UGT2B17 glucuronidation activity.

## Discussion

Sex disparity in the development of hematological malignancies has been well documented. A genomic basis for these differences is in the occurrence of somatic mutations on the sex chromosomes [7]. Of the six genes for which a sex bias was found in different tumor entities [7], only *DDX2X* had been reported previously in the context of poor risk CLL and clonal evolution [30, 31], Considering, however, the importance of sex steroids and gonadotropins for development and function of the immune system [6], we decided to focus our study on hormones and hormone metabolites.

We report a comprehensive profiling of circulating sex steroids and pituitary hormones in men and women with CLL, revealing a sex-specific association of these signaling molecules to treatment-free survival. We observed that high levels of potent androgens and biologically active estrogen metabolites are linked to an improved survival of female CLL patients whereas higher LH levels are associated with shorter survival in male CLL patients. These observations support that CLL is a hormone-responsive disease and imply different biological mechanisms associated with progression of leukemia in men and women.

We observed a significant association of high levels of testosterone and DHT, and improved TFS in women suggesting that the AR signaling axis may confer beneficial effects, potentially delaying disease progression, whether directly or indirectly acting on leukemic cells. This relationship remained similar after adjustment for prognostic factors, suggesting that effect of androgens is not dependent on these molecular changes. Testosterone is one of very few steroids present at higher concentrations in circulation of women with CLL compared to healthy postmenopausal donors, despite lower levels of adrenal precursors, suggesting a potential influence of the disease on androgen synthesis. Little is known regarding the expression and function of the AR in CLL and this information would be essential to provide a better understanding of the potential impact of androgen signaling on CLL progression. While data denoted that androgens have immunosuppressive properties [5], some of these cellular processes may be relevant to CLL progression including the alteration of bone marrow stromal cell behavior and responses to small molecular weight mediators such as cytokines [32, 33]. In line, recent reports suggested potential therapeutic or prognostic applications to targeting the AR axis in other lymphoid malignancies such as mantle cell lymphoma and diffuse large B cell lymphoma [13, 34].

An alternative hypothesis to our observation in women with CLL may involve the aromatization of testosterone to estradiol, an efficient agonist for ERβ, which is the main ER expressed in lymphoid tissues also overexpressed in CLL [11, 15, 35]. Accordingly, the improved TFS in female cases may be, at least in part, the consequence of a greater local exposure of leukemic cells to estrogens, described as tumor-suppressive molecules acting via the ER axis in cells of lymphoid origin that express ERβ [36]. Concurring with this hypothesis, treatment with aromatase inhibitors blocking the conversion of androgen to estrogen was shown to enhance lymphoma growth in mice, but not androgens per se [37]. Also, activation of ERβ was shown to induce autophagy and impair cell proliferation of Hodgkin lymphoma [38] while ERβ1 nuclear expression was exposed as an independent prognostic factor for adverse progression-free survival in diffuse large B cell lymphoma (DLBCL) cases [39, 40].

We also observed that higher levels of methoxylated estrogen metabolites are associated with prolonged TFS in female CLL patients compared to those with lower levels. MeO-CE corresponds to abundant endogenous estrogen derivatives that cause growth arrest of hormone-dependent and hormone-independent tumors in vitro and in vivo, consistent with their protective effect observed in female CLL cases [41]. Several studies have shown that MeO-CE exerts anticarcinogenic, antiproliferative, antiangiogenic, pro-apoptotic, and anti-inflammatory properties [42, 43]. This is in opposition to their mitogenic hydroxylated counterparts (2/4OH-CE) that can be metabolized into quinones leading to the formation of quinone adducts and oxidative DNA damage [17]. These reactive estrogen metabolites are critical in the initiation of breast and prostate cancers as well as non-Hodgkin lymphoma, and have been found in abundance in leukemia cell lines of the NCI-60 human tumor cell lines panel [44, 45]. This may indicate that increased methylation of estrogens could be beneficial for CLL patients by preventing the formation and accumulation of damaging catechol estrogen metabolites and/or through their intrinsic beneficial effects. In support of the later, 2MeOE_2_ was recently shown to abrogate preleukemic stem cell self-renewal when maintained in a niche-like environment, inducing apoptosis and exhibiting antileukemic activity in primary human T cell acute lymphoblastic leukemia blasts [46] concurring with the protective association for MeO-CE observed herein.

It may not be surprising to observe a more pronounced adverse effect associated with high mRNA expression of the steroid-inactivating UGT2B17 enzyme in leukemic cells of female patients, where a higher systemic exposure to its androgenic substrates is linked to beneficial effects on disease progression. Besides, we could verify the ability of the UGT2B17 enzyme to conjugate MeO-CE in vitro (not shown). This is supported by our observation of higher levels of MeO-CE in female CLL cases with the UGT2B17^null^ genotype compared to those carrying the gene, despite the small sample size. In turn, no differences were apparent in male CLL cases notwithstanding similar circulating concentrations of MeO-CE. This may reflect sex differences in the metabolic enzymes involved in the conversion of androgen to estrogen and their subsequent biotransformation to MeO-CE by catechol-*O*-methyltransferase (COMT). It is thus conceivable that *UGT2B17* mRNA expression in CLL modifies exposure of leukemic cells and/or proliferation centers to these steroids with a resulting alteration of leukemic cell behavior or of the microenvironment.

For male CLL patients, we observed an association between higher levels of LH and shorter TFS. An earlier study reported higher levels of LH in male CLL patients compared to controls and an association to Rai stage [47], suggesting that men with higher levels of LH correspond to progressive cases. LH pituitary secretion is regulated by testosterone levels that decline with age with a corresponding increase in LH in aging men [48]. We did not observe a correlation between levels of these two circulating hormones in male CLL cases (*r* = 0.12; *P* = 0.30), denoting that LH may represent a biomarker in CLL. As for the underlying mechanism, a recent study presented evidence that pituitary gonadotropins may act on lymphocyte migration and that leukemia cells recognize these hormones as chemoattractants [49]. Hematopoietic stem cells have been shown to express nearly all hormone receptors, but it is unknown whether their expression is maintained through maturation and differentiation [50]. Evidence for the expression of LH receptor in mature B lymphocytes and CLL cells is still lacking.

Among the strengths of the study are the abundance of available clinical and molecular data as well as the wide range of hormones that are reported for the first time in CLL patients. TFS was retained as the primary endpoint since it is disease-specific and not limited by competing risks of death by other conditions. In addition, whereas previous studies used radioimmunoassays to measure sex steroids, we used validated MS assays that are more specific, sensitive, and accurate [27]. Our cohort of CLL patients was composed of aged men and postmenopausal women. We excluded premenopausal women to limit confounding factors such as menstrual cycle that largely affects circulating steroid levels. Some limitations of our study, exploratory in nature, also need to be considered. Body mass index was not recorded and may have affected the relationship between disease progression and steroid levels considering the association of body mass index with circulating steroids [51].

Sex differences in cytogenetic anomalies, as reported previously [4], were not statistically significant in this study cohort. We also explored potential differences in circulating hormone levels in CLL patients compared to those of healthy subjects revealing hormonal imbalances with the onset of CLL and raising the possibility that the disease may affect hormone synthesis and/or metabolism. The variations observed are unlikely to be linked to molecular events commonly associated with CLL, such as gene deletions or IGHV status, as no obvious association between these variables and hormone levels was noted (not shown).

This study reports a sex-specific hormonal imbalance and association between circulating sex steroids and pituitary hormones and treatment-free survival in CLL patients. Larger studies will need to be conducted to replicate our initial observations and to assess potential changes in hormone levels during the evolution of the disease and how drug treatment potentially affects their relationship with disease progression, both in men and women. Further work is also required to elucidate whether these effects are mediated by leukemic cells and/or affecting the dynamic interactions with the microenvironment in order to identify mediators and signaling pathways involved. Our work creates opportunities for additional studies on the role of sex steroids and pituitary hormones that may participate in autocrine/paracrine loops affecting the survival and proliferation of CLL cells.

## Electronic supplementary material

ESM 1 (PDF 400 kb)

## Abbreviations

Testo: testosterone
DHT: dihydrotestosterone
4-dione: 4-androstenedione
DHEA: dehydroepiandrosterone
DHEA-S: dehydroepiandrosterone sulfate
5-diol: 5-androsten-3β, 17β-diol
E_1_: estrone
E_1_-S: estrone sulfate
E_2_: estradiol
ADT: androsterone
ADT-G: androsterone glucuronide
Prog: progesterone
3β-diol-androstane: 3β-17β-diol
3α-diol-17G-androstane: 3α, 17β-diol-17-glucuronide
3α-diol-3G-androstane: 3α, 17β-diol 3-glucuronide
E_3_: estriol
16epiE_3_: 16-epiestriol
16ketoE_2_: 16-ketoestradiol
16αOHE_1_: 16α-hydroxyestrone
2MeOE_1_: 2-methoxyestrone
4MeOE_1_: 4-methoxyestrone
2OHE_1_: 2-hydroxyestrone
4OHE_1_: 4-hydroxyestrone
CE: catechol estrogen
2/4OH-CE: hydroxy catechol estrogen
16OH-CE: 16-hydroxy catechol estrogen
MeO-CE: methoxy catechol estrogen
CI: confidence interval
CLL: chronic lymphocytic leukemia
CV: coefficient of variation
TFS: treatment-free survival
VH: immunoglobulin variable heavy chain
HR: hazard ratio
