# Supplementary material for: Sex-dependent association of circulating sex steroids and pituitary hormones with treatment-free survival in chronic lymphocytic leukemia patients
Source: Ann Hematol. 2018 May 21;97(9):1649–61. doi: 10.1007/s00277-018-3356-z (PMC6097785; doi:10.1007/s00277-018-3356-z)
Supplement: Supplementary file 1 — (PDF 400 kb) [file 277_2018_3356_MOESM1_ESM.pdf]

Supplementary Material

1. Supplementary Methods

1.1 Mass spectrometry-based quantification of steroids.

2. Supplementary Tables

2.1 Supplementary Table 1 Circulating hormones in men and women CLL patients

2.2 Supplementary Table 2 Circulating steroid levels of men with CLL compared to those of healthy individuals

2.3 Supplementary Table 3 Circulating steroid levels of women with CLL compared to those of healthy individuals

2.4 Supplementary Table 4 Association of high or low *UGT2B17* mRNA expression in peripheral mononuclear blood cells (PBMCs) with treatment-free survival (TFS) in CLL patients (male and female combined)

2.5 Supplementary Table 5 Treatment free survival (TFS) is not significantly affected he *UGT2B17* deletion polymorphism

2.6 Supplementary Table 6 Circulating hormone levels of CLL patients in relation to the *UGT2B17* deletion polymorphism

1. Methods

1.1 Mass spectrometry-based quantification of steroids

Steroids were purchased from Steraloids (Newport, RI, USA). Internal deuterated standards were added to samples. Deuterated standards were from C/D/N Isotopes (Montréal, QC, Canada), except d3-DHEA, which was synthesized by the Organic Synthesis Service of the CHU de Québec Research Center (Québec, QC, Canada). Quality controls were prepared in non-adsorbed serum samples to obtain low, medium or high analyte concentrations and were included in each run, along with a seven-point calibration curve prepared by spiking, as well as blanks.

For catechol estrogen (reported as the sum of conjugated plus unconjugated forms), we used 250 µL of serum for extraction with a LLOQ of 5 pg/mL (ratio of signal-to-noise was ≥5:1 corresponding to 16.56-18.52 pmol/L depending on the estrogen). Samples were treated with β-glucuronidase/sulfatase prior to extraction with ethyl acetate:chlorobutane (25:75, v/v) and evaporated to dryness. Derivatization was then conducted with dansyl chloride (0.5 mg/mL final in 50% acetone and 50 mM sodium bicarbonate, pH 9.0). Samples were heated for 5 minutes at 60°C, mixed with 15 volumes of water:methanol (80:20, v/v) and loaded on pre-conditioned Strata X 60 mg SPE columns (Phenomenex, Torrance, CA, USA). After being washed with water and water:methanol (10:90, v/v), CE were eluted with dichloromethane:methanol (50:50, v/v) and evaporated to dryness at 45°C under nitrogen gas, reconstituted in 100 µL of acetone:water (75:25, v/v), and injected into a HPLC Waters alliance 2690 (Milford, MA, USA). The chromatographic separation was achieved with a Synergie RP Hydro column containing 2.5 µm packing material, 100 X 3 mm (Phenomenex, Torrance, USA). The mobile phases consisted of water with 0.0375% formic acid (solvent A) and MeOH with 0.0375% formic acid (solvent B). The flow rate was 0.5 ml/min with the following program: 0-8 min, isocratic 22.5% B; 8-18 min, linear gradient 22.5-35% B; 18-23 min, isocratic 35% B; 23-23.1 min, linear

gradient 35-95% B; 23.1-28 min, isocratic 95% B; 28.0-28.1 min, linear gradient 95-22.5% B and 28.1-33 min, isocratic 22.5% B. CE were detected with an API6500 (Concord, ON, Canada) equipped with a turbo ion-spray source set in positive ion mode, and operated in multiple reaction monitoring mode (MRM).

2. Supplementary Tables

2.1 Supplementary Table 1 Circulating hormones in men and women CLL patients.

Plasma steroid levels	Men (n=95)	Women (n=61)	Ratio
	Mean ± SEM		Men/Women
**Adrenal precursors**			
DHEA-S (µg/mL)	0.75 ± 0.07	0.49 ± 0.05	**1.5**
DHEA (ng/mL)	1.35 ± 0.12	1.56 ± 0.15	0.9
5-diol (pg/mL)	577.21 ± 37.01	384.21 ± 36.48	**1.5**
**Androgens**			
4-dione (ng/mL)	0.82 ± 0.03	0.52 ± 0.04	**1.6**
Testo (ng/mL)	3.86 ± 0.20	0.26 ± 0.06	**15.1**
DHT (pg/mL)	293.86 ± 17.63	36.18 ± 6.13	**8.0**
ADT (pg/mL)	140.06 ± 9.01	93.85 ± 8.94	**1.4**
3β-diol (pg/mL)	18.72 ± 1.36	7.68 ± 0.83	**2.3**
ADT-G (ng/mL)	29.72 ± 2.09	13.37 ± 1.34	**2.2**
3α-diol-17G (ng/mL)	3.36 ± 0.28	0.40 ± 0.06	**8.4**
3α-diol-3G (ng/mL)	1.63 ± 0.13	0.60 ± 0.05	**2.7**
**Estrogen**			
E_1-S (ng/mL)	0.43 ± 0.04	0.15 ± 0.02	**2.9**
E_1 (pg/mL)	26.59 ± 1.52	16.80 ± 1.26	**1.6**
E_2 (pg/mL)	17.33 ± 0.86	3.37 ± 0.52	**4.9**
**Receptor ligands***			
ER-ligands (pg/mL)	639.86 ± 38.00	411.35 ± 37.49	**1.6**
AR-ligands (ng/mL)	4.15 ± 0.21	0.29 ± 0.07	**14.3**
**Catechol estrogens (CE)†**	(n=83)	(n=51)	
2/4OH-CE (pg/mL)	55.08 ± 5.69	36.25 ± 6.80	**1.5**
16OH-CE (pg/mL)	205.46 ± 25.98	103.62 ± 25.59	**2.0**
MeO-CE (pg/mL)	38.90 ± 2.04	32.92 ± 2.29	**1.2**
**Pituitary gonadotropins**	(n=83)	(n=51)	
LH (mIU/mL)	6.63 ± 0.68	17.49 ± 1.21	**0.4**
FSH (mIU/mL)	11.94 ± 1.11	49.03 ± 2.32	**0.2**

Significant (P<0.05) ratios are in bold; trends (P<0.10) are in italics, based on Mann-Whitney-Wilcoxon test. Hormone levels for all cases were available, except catechol estrogens and gonadotropins (134/156); SEM - standard error of the mean.

*ER-ligands corresponds to the sum of E_1, E_2, 5-diol, 3β-diol; AR-ligands corresponds to the sum of Testo and DHT. ER - estrogen receptor; AR = androgen receptor.

†2/4OH-CE corresponds to the sum of $2OHE_1$ and $4OHE_1$. 16OH-CE corresponds to the sum of E_3, $16epiE_3$, $16ketoE_2$, and $16\alpha OHE_1$. Sum of MeO-CE corresponds to the sum of $2MeOE_1$ and $4MeOE_1$. CE - catechol estrogens.

In men, a partial negative correlation was noted between LH and FSH and levels of DHEA-S, DHEA, ADT and E_1-S (correlation values of r = -0.22 to -0 51; P<0.05). In women, a partial positive correlation was noted between LH and levels of ADT-G (correlation values of r = -0.22 to -0 51; P=0.007).

2.2 Supplementary Table 2 Circulating steroid levels of men with CLL compared to those of healthy individuals.

**Plasma steroid levels**	**Men CLL cases** Austria (n=95)	**Healthy men** Austria (n=5)	**Ratio** C/H	**Healthy men** Canada (n=15)	**Ratio** C/H
	Mean ± SEM			Mean ± SEM	
**Adrenal precursors**					
DHEA-S (µg/mL)	0.75 ± 0.07	1.71 ± 0.44	**0.44**	1.96 ± 0.22	**0.38**
DHEA (ng/mL)	1.35 ± 0.12	5.79 ± 1.70	**0.23**	4.84 ± 0.75	**0.28**
5-diol (pg/mL)	577.21 ± 37.01	1186.57 ± 445.38	0.49	1155.36 ± 115.59	**0.50**
**Androgens**					
4-dione (ng/mL)	0.82 ± 0.03	1.53 ± 0.39	*0.54*	1.22 ± 0.11	**0.67**
Testo (ng/mL)	3.86 ± 0.20	5.11 ± 1.26	0.75	4.80 ± 0.35	**0.80**
DHT (pg/mL)	293.86 ± 17.63	381.42 ± 94.35	0.77	358.92 ± 34.17	0.82
ADT (pg/mL)	140.06 ± 9.01	273.47 ± 58.03	**0.52**	257.18 ± 23.90	**0.55**
3β-diol (pg/mL)	18.72 ± 1.36	18.56 ± 7.25	1.02	46.53 ± 4.66	**0.41**
ADT-G (ng/mL)	29.72 ± 2.09	36.66 ± 8.18	0.81	55.18 ± 6.65	**0.54**
3α-diol-17G (ng/mL)	3.36 ± 0.28	2.79 ± 0.48	1.21	4.38 ± 0.53	**0.77**
3α-diol-3G (ng/mL)	1.63 ± 0.13	1.25 ± 0.29	1.30	2.43 ± 0.34	**0.67**
**Estrogens**					
E_1-S (ng/mL)	0.43 ± 0.04	0.43 ± 0.17	1.01	0.43 ± 0.11	0.99
E_1 (pg/mL)	26.59 ± 1.52	29.27 ± 1.83	0.91	22.51 ± 2.03	1.18
E_2 (pg/mL)	17.33 ± 0.86	19.72 ± 2.64	0.88	19.56 ± 2.01	0.89
**Receptor ligands***					
ER-ligands (pg/mL)	639.86 ± 38.01	1254.12 ± 450.76	0.51	1243.96 ± 118.21	**0.51**
AR-ligands (ng/mL)	4.15 ± 0.21	5.49 ± 1.35	0.76	5.15 ± 0.36	**0.81**
**Catechol estrogens (CE)†**	(n=83)	(n=5)			
2/4OH-CE (pg/mL)	55.08 ± 5.69	41.62 ± 14.13	1.32	-	
16OH-CE (pg/mL)	205.46 ± 25.98	78.52 ± 6.41	*2.62*	-	
MeO-CE (pg/mL)	38.90 ± 2.04	37.31 ± 7.86	1.04	-	
**Pituitary gonadotropins**	(n=83)	(n=5)		(n=12)	
LH (mIU/mL)	6.63 ± 0.68	2.49 ± 0.72	**2.66**	4.72 ± 0.56	1.40
FSH (mIU/mL)	11.94 ± 1.11	3.12 ± 0.62	**3.83**	5.57 ± 1.39	**2.14**

Significant (P<0.05) ratios are in bold, trends (P<0.10) are in italics, based on Mann-Whitney-Wilcoxon test. Hormone levels for all cases were available, except catechol estrogens and gonadotropins (134/156); SEM - standard error of the mean. Ratio C/H = ratio between levels observed in cases/healthy.

*ER-ligands corresponds to the sum of E_1, E_2, 5-diol, 3β-diol; AR-ligands corresponds to the sum of Testo and DHT. ER - estrogen receptor; AR = androgen receptor.

†2/4OH-CE corresponds to the sum of $2OHE_1$ and $4OHE_1$. 16OH-CE corresponds to the sum of E_3, $16epiE_3$, $16ketoE_2$, and $16\alpha OHE_1$. Sum of MeO-CE corresponds to the sum of $2MeOE_1$ and $4MeOE_1$. CE - catechol estrogens.

2.3 Supplementary Table 3 Circulating steroid levels of women with CLL compared to those of healthy individuals.

Plasma steroid levels	**Women CLL cases** Austria (n=61) Mean ± SEM	**Healthy women** Austria (n=5) Mean ± SEM	**Ratio** C/H	**Healthy women** Canada (n=110) Mean ± SEM	**Ratio** C/H
**Adrenal precursors**					
DHEA-S (µg/mL)	0.49 ± 0.05	0.94 ± 0.28	*0.52*	0.70 ± 0.04	**0.70**
DHEA (ng/mL)	1.56 ± 0.15	2.66 ± 0.69	*0.59*	2.25 ± 0.14	**0.69**
5-diol (pg/mL)	384.21 ± 36.48	225.58 ± 86.40	1.70	259.09 ± 15.58	**1.48**
**Androgens**					
4-dione (ng/mL)	0.52 ± 0.04	0.50 ± 0.07	1.05	0.48 ± 0.02	1.10
Testo (ng/mL)	0.26 ± 0.06	0.23 ± 0.05	1.13	0.15 ± 0.01	**1.67**
DHT (pg/mL)	36.18 ± 6.13	39.28 ± 8.43	0.94	36.36 ± 2.14	1.01
ADT (pg/mL)	93.85 ± 8.94	123.51 ± 20.86	0.83	-	
3β-diol (pg/mL)	7.68 ± 0.83	9.82 ± 3.15	0.82	-	
ADT-G (ng/mL)	13.37 ± 1.34	16.97 ± 6.98	0.79	15.21 ± 0.89	*0.88*
3α-diol-17G (ng/mL)	0.40 ± 0.06	0.46 ± 0.10	0.87	0.73 ± 0.09	**0.54**
3α-diol-3G (ng/mL)	0.60 ± 0.05	0.71 ± 0.13	0.85	0.67 ± 0.06	0.91
**Estrogen**					
E_1-S (ng/mL)	0.15 ± 0.02	0.46 ± 0.27	0.32	0.25 ± 0.03	**0.58**
E_1 (pg/mL)	16.80 ± 1.26	22.87 ± 4.54	0.73	21.14 ± 1.20	**0.79**
E_2 (pg/mL)	3.37 ± 0.52	10.99 ± 5.98	0.32	5.86 ± 1.00	**0.60**
**Receptor ligands**					
ER-ligands (pg/mL)	411.35 ± 37.49	267.29 ± 93.43	1.54	286.08 ± 16.19	1.44
AR-ligands (ng/mL)	0.29 ± 0.07	0.27 ± 0.06	1.10	0.19 ± 0.01	1.55
**Catechol estrogens (CE)**	(n=51)	(n=5)			
2/4OH-CE (pg/mL)	36.25 ± 6.80	30.96 ± 11.58	1.17	-	
16OH-CE (pg/mL)	103.62 ± 25.59	58.97 ± 14.70	1.76	-	
MeO-CE (pg/mL)	32.92 ± 2.29	31.92 ± 5.39	1.03	-	
**Pituitary gonadotropins**	(n=51)	(n=5)		(n=110)	
LH (mIU/mL)	17.49 ± 1.21	25.47 ± 3.41	**0.67**	25.33 ± 0.33	**0.69**
FSH (mIU/mL)	49.03 ± 2.32	72.57 ± 8.43	**0.68**	66.35 ± 2.01	**0.74**

Significant (P<0.05) ratios are in bold, trends (P<0.10) are in italics, based on Mann-Whitney-Wilcoxon test. Hormone levels for all cases were available, except catechol estrogens and gonadotropins (134/156); SEM - standard error of the mean. Ratio C/H = ratio between levels observed in cases/healthy.

*ER-ligands corresponds to the sum of E_1, E_2, 5-diol, 3β-diol; AR-ligands corresponds to the sum of Testo and DHT. ER - estrogen receptor; AR = androgen receptor.

†2/4OH-CE corresponds to the sum of $2OHE_1$ and $4OHE_1$. 16OH-CE corresponds to the sum of E_3, $16epiE_3$, $16ketoE_2$, and $16\alpha OHE_1$. Sum of MeO-CE corresponds to the sum of $2MeOE_1$ and $4MeOE_1$. CE - catechol estrogens.

- : not available

2.4 Supplementary Table 4 Association of high or low *UGT2B17* mRNA expression in peripheral mononuclear blood cells (PBMCs) with treatment-free survival (TFS) in CLL patients (male and female combined).

	**UGT2B17-high** % (n=66)	**UGT2B17-low** % (n=87)
**TFS, median (mo)**	**75.5**	**126.3**
**Requiring treatment**	**69.7% (n=46)**	**51.2% (n=45)**

Significant (*P*<0.05) ratios are in bold, trends (*P*<0.10) are in italics. Calculated using the Kaplan-Meier method.

2.5 Supplementary Table 5 Treatment free survival (TFS) is not significantly affected he *UGT2B17* deletion polymorphism.

**Men (n=95)** Median TFS (months)		**Women (n=60)[1]** Median TFS (months)	
0 copy (n=14)	1-2 copies (n=81)	0 copy (n=9)	1-2 copies (n=49)
61.9	82.4	*254*	126

Significant (*P*<0.05) differences are in bold, trends (*P*<0.10) are in italics, based on the Log-rank test. Part of these data was included in the UGT2B17 study by Gruber et al.[18] the information was complemented and updated for this study cohort.

0 copy = UGT2B17$^{del/del}$ or UGT2B17^{null} genotype; 1-2 copies = patients carrying at least one copy of the *UGT2B17* gene.

[1]60 out of 61 women had UGT2B17 copy number information.

2.6 Supplementary Table 6 Circulating hormone levels of CLL patients in relation to the *UGT2B17* deletion polymorphism.

Plasma steroid levels	Men CLL cases (n = 95)		Women CLL cases (n = 60)‡	
	0 copy (n=14)	1-2 copies (n=81)	0 copy (n=9)	1-2 copies (n=51)
	Mean ± SEM		Mean ± SEM	
**Adrenal precursors**				
DHEA-S (µg/mL)	0.83 ± 0.21	0.74 ± 0.07	0.40 ± 0.11	0.49 ± 0.05
DHEA (ng/mL)	1.75 ± 0.62	1.28 ± 0.09	1.38 ± 0.27	1.53 ± 0.16
5-diol (pg/mL)	602.11 ± 71.25	572.91 ± 41.76	330.48 ± 11.08	383.36 ± 38.78
**Androgens**				
4-dione (ng/mL)	*0.98 ± 0.09*	*0.79 ± 0.04*	0.51 ± 0.74	0.52 ± 0.04
Testo (ng/mL)	*4.69 ± 0.51*	*3.71 ± 0.21*	0.19 ± 0.03	0.26 ± 0.04
DHT (pg/mL)	381.91 ± 65.47	278.64 ± 16.99	28.77 ± 4.83	36.91 ± 7.26
ADT (pg/mL)	160.84 ± 22.47	136.47 ± 9.83	106.83 ± 17.92	88.89 ± 9.86
3β-diol (pg/mL)	19.69 ± 2.52	18.55 ± 1.54	9.71 ± 2.78	7.38 ± 0.87
ADT-G (ng/mL)	29.97 ± 5.16	29.67 ± 2.30	12.81 ± 3.84	13.03 ± 1.39
3α-diol-17G (ng/mL)	2.79 ± 0.45	3.46 ± 0.32	**0.15 ± 0.02**	**0.44 ± 0.07**
3α-diol-3G (ng/mL)	1.40 ± 0.17	1.67 ± 0.16	0.50 ± 0.14	0.61 ± 0.06
**Estrogens**				
E_1-S (ng/mL)	0.54 ± 0.12	0.41 ± 0.04	0.16 ± 0.06	0.14 ± 0.02
E_1 (pg/mL)	28.84 ± 3.63	26.20 ± 1.67	19.84 ± 4.55	15.92 ± 1.23
E_2 (pg/mL)	19.65 ± 1.79	16.93 ± 0.95	3.07 ± 0.90	3.30 ± 0.60
**Receptor ligands***				
ER-ligands (pg/mL)	670.29 ± 71.14	634.53 ± 42.97	363.11 ± 99.13	408.34 ± 40.16
AR-ligands (ng/mL)	*5.07 ± 0.57*	*3.99 ± 0.23*	0.22 ± 0.03	0.30 ± 0.08
**Catechol estrogens (CE)†**	(n=14)	(n=69)	(n=9)	(n=42)
2/4OH-CE (pg/mL)	48.25 ± 7.87	56.46 ± 6.66	35.10 ± 6.30	36.53 ± 8.18
16OH-CE (pg/mL)	154.84 ± 25.14	215.73 ± 30.75	239.86 ± 128.33	74.43 ± 12.77
MeO-CE (pg/mL)	40.17 ± 5.12	38.64 ± 2.24	35.88 ± 6.81	32.28 ± 2.41
**Pituitary gonadotropins**	(n=14)	(n=69)	(n=9)	(n=42)
LH (mIU/mL)	5.46 ± 1.00	6.86 ± 0.79	17.70 ± 3.64	17.44 ± 1.28
FSH (mIU/mL)	7.87 ± 1.34	12.77 ± 1.29	42.56 ± 5.51	50.42 ± 2.54

UGT2B17 copy number variation is described as 0 = UGT2B17$^{del/del}$; 1-2 copies = patients carrying at least one copy of the gene. Frequency of UGT2B17$^{del/del}$ or UGT2B17^{null} genotype was of 14.7% and 15.0% in male and female cases respectively. Significant (P<0.05) ratios are in bold, trends (P<0.10) are in italics, based on Mann-Whitney-Wilcoxon test. Hormone levels for all cases were available, except catechol estrogens and gonadotropins (134/156); SEM - standard error of the mean. Ratio C/H = ratio between levels observed in cases/healthy.

*ER-ligands corresponds to the sum of E_1, E_2, 5-diol, 3β-diol; AR-ligands corresponds to the sum of Testo and DHT. ER - estrogens receptor; AR = androgen receptor.

†2/4OH-CE corresponds to the sum of $2OHE_1$ and $4OHE_1$. 16OH-CE corresponds to the sum of E_3, $16epiE_3$, $16ketoE_2$, and $16\alpha OHE_1$. Sum of MeO-CE corresponds to the sum of $2MeOE_1$ and $4MeOE_1$. CE - catechol estrogens.

‡The *UGT2B17* deletion genotype was missing for one woman.
